# Effect of the Incorporation of an Innovative Monomer with a Quaternary Ammonium Group into a Temporary Soft Liner on Its Biological and Physicochemical Properties

**DOI:** 10.3390/molecules30040941

**Published:** 2025-02-18

**Authors:** Patrycja Kula, Izabela Barszczewska-Rybarek, Anna Mertas, Grzegorz Chladek

**Affiliations:** 1 Department of Physical Chemistry and Technology of Polymers, Faculty of Chemistry, Silesian University of Technology, Strzody 9 Str., 44-100 Gliwice, Poland; patrycja.kula@polsl.pl (P.K.); izabela.barszczewska-rybarek@polsl.pl (I.B.-R.); 2 Department of Microbiology and Immunology, Faculty of Medical Sciences in Zabrze, Medical University of Silesia in Katowice, 19 Jordana Str., 41-808 Zabrze, Poland; amertas@sum.edu.pl; 3 Materials Research Laboratory, Faculty of Mechanical Engineering, Silesian University of Technology, Konarskiego 18A Str., 44-100 Gliwice, Poland

**Keywords:** temporary soft lining, quaternary ammonium, antifungal properties, mechanical properties, physicochemical properties, cytotoxicity, dental materials, *Candida albicans*

## Abstract

The colonizing of temporary soft lining materials in the oral cavity by yeast-like fungi, particularly *Candida albicans*, poses a significant risk of complications during prosthetic treatment. Various experimental materials incorporating antimicrobial additives, such as drugs, natural oils, and inorganic particles, have been tested. However, these components are not chemically bonded to a polymer network, making them prone to being easily released into the surrounding environment. This study aimed to evaluate experimental soft lining materials containing liquid components with 2-(methacryloyloxy)ethyl-2-decylhydroxyethylmethylammonium bromide, a monomethacrylate monomer with a quaternary ammonium group, added at concentrations of 8.54%, 8.75%, and 14.90% by weight. The adherence of *Candida albicans*, cytotoxicity, glass transition temperature (*Tg*), sorption (*WS*), solubility (*WSL*), Shore A hardness (*SHA*), tensile strength (*TS*), and tensile bond strength (*TBS*) were tested. Two tested materials did not show cytotoxicity for the 2-day undiluted extracts. The *Candida albicans* adhesions were reduced for two materials. The *SHA* values compared to the control were varied but all decreased with time. *WS* and *WSL* increased compared to the control. The *TBS* values were at an acceptable level.

## 1. Introduction

In the human oral cavity, approximately 700 species of microorganisms, such as yeasts, fungi, and bacteria, coexist in symbiosis with each other and the host [1]. Specific microenvironmental conditions also favor biofilm formation, its adhesion to oral tissues and prosthetic devices, and the growth of microorganisms in them [2]. An important part of the oral microflora are mycoflora such as *Candida* spp., which occur in 30–65% of the healthy population [3]. However, the emergence of favorable conditions can lead to the pathogenesis of *Candida* spp., called invasive or systemic candidiasis, or less frequently candidemia [4]. Patients during different stages of dental treatments are particularly vulnerable to health problems related to the presence of *Candida* spp., especially *Candida albicans* (*C. albicans*), due to the high risk of soft tissue injuries, the properties of dental materials and favorable microenvironmental conditions, and the growth of pathogenic microflora (as a result of high temperatures, high humidity, a low pH value, and a closed space between soft tissue and the denture) [5]. Furthermore, colonization of the oral cavity or prosthetic devices by *Candida* spp. also increases the threat of *Candida* spp. transport through the bloodstream [6], inhalation, and/or swallowing and complications, thus affecting the risk of the infection of the kidneys, heart, brain, lungs, and liver [6,7].

Temporary lining materials are crosslinked plasticized acrylates used in dental practice for a period of up to 30 days to reline immediate dentures, for the wound healing of mucosa, for example, after surgical procedures or for denture function, but also in cases of mucosa pain caused by the hard denture or to temporarily improve the fit of the prosthesis to improve stabilization and prevent injuries [8,9,10]. Preventing injuries and facilitating wound healing reduces the risk of complications associated with possible tissue infection by oral microorganisms such as *Candida* spp. The protective role of these materials is related to their low modulus of elasticity and ability to dissipate energy during chewing [11]. However, these hydrophilic materials themselves are susceptible to rapid colonization and even penetration by pathogenic microorganisms [12,13], which can limit the effectiveness of treatment with their use [14]. Moreover, surface microdamage created by the use of denture cleaning agents can be a cause of increased adherence and the rapid recolonization of linings by yeast-like fungi [15], so developing the antimicrobial properties of a temporary relining is considered one of the principal development paths of these materials.

Reports emphasize the role of the necessary disinfection of dental prostheses by immersing them in solutions of compounds that exhibit antibacterial and antifungal activity to reduce the number of adhered colonies of microorganisms on the surface of the dentures; however, this cannot resolve the problem due to their limited durability and effectiveness [16,17], which is associated inter alia with the penetration of microbial cells into the interior of the material [18] or their survival in micro-cracks on the surface [19] that are inaccessible to cleaning fluids. One of the promising strategies to reduce *Candida*-related complications is developing acrylic SLMs with antifungal properties by introducing antimicrobial compounds. Ingredients that can damage the cell membrane or modify DNA and RNA [20], such as polyene antibiotics [21], azole drugs [22], organic [23,24] or inorganic [25,26] nanoparticles, or plant extracts and oils [27,28], have been considered. However, the mentioned antimicrobial chemical compounds cannot copolymerize with the methacrylate monomers included in acrylic SLMs; therefore, they tend to uncontrolled release from the materials, so they do not provide stable antimicrobial resistance [29]. In addition, the use of antibiotics is criticized due to the increasing resistance of microorganisms [29], while the risk of the compounding of nano-additives that can reach other parts of the body by swallowing and the respiratory tract is only partially recognized [30,31,32].

A proposal to solve this problem may be the covalent introduction of antimicrobial monomers. The quaternary ammonium group (QAG) shows high antimicrobial activity [33], so the synthesis of monomers that contain it and one or more methacrylate groups creates the ability of copolymerization with other methacrylate components included in dental acrylates. Through copolymerization, the risk of the release of introduced active components can be minimalized, ensuring the stability of its antimicrobial properties [34]. This concept was first proposed and tested for use in materials for reconstructive dentistry. It involves copolymerizing mono- or dimethacrylate monomers containing one or more quaternary ammonium groups with dental composite resins. Monomethacrylates were the first group of monomers to be studied, which can be represented by methacryloxydodecylpyridinium bromide (MDPB) [35]. Further research on monomethacrylate monomers was focused on those based on 2-dimethylaminoethyl methacrylate (DMAEMA), with the quaternary nitrogen substituted with various alkyl chains. Dimethacrylates that contain two quaternary ammonium groups derived from DMAEMA include the following: hexadecyl methyl ammonium bromide; 2-methacryloxyethyl dodecyl methyl ammonium bromide (MAE-DB); N, N’-bis[2-(methacryloxyloxy) ethyl] N, N, N’, N’ tetramethyl N.N’ butanediyl diammonium bromide (DMBB); and N, N’-bis[2-(methacryloxyloxy) ethyl] N, N, N’, N’ tetramethyl N, N’ hexanediyl diammonium bromide (DMBH) [36,37,38]. Quaternized derivatives of Bis-GMA [39] and UDMA monomer [40] were also synthesized. All the obtained copolymers showed antibacterial activity against Gram-positive (such as *Streptococcus mutans* and *Staphylococcus aureus*) and Gram-negative (such as *Escherichia coli*) bacteria. Occasionally, similar solutions were tested for antifungal activity, and this also revealed promising properties for light-cured bis-GMA-based polymers [41] and polymethyl methacrylate (PMMA) denture base resin [42]. However, until now there have been no studies on the introduction of this type of monomer into plasticized, elastic soft lining materials (SLMs). It is important to note that, unlike composite resins or PMMA denture base resins, a low modulus of elasticity and hardness are required in this case. In this study, we present an innovative approach to SLMs, developed through the abovementioned idea of chemical modification with antimicrobial monomers. In this research, we present an innovative approach to temporary SLMs by chemical modification with antimicrobial monomers. Therefore, this study aimed to investigate the antimicrobial, immunological, and physicomechanical properties of experimental acrylate SLMs that contain a new synthesized monomethacrylate monomer with introduced QAG, 2-(methacryloyloxy)ethyl-2-decylhydroxyethylmethylammonium bromide (QAHAMA-C10). The thesis was that it is possible to obtain acrylic SLMs for temporary use by the introduction of QAHAMA-C10 monomer with QAG that is characterized by the decreased antifungal adherence of *C. albicans* and biofunctional properties suitable for SLM.

## 2. Results

### 2.1. Monomer Characterization

**Figure 1:**
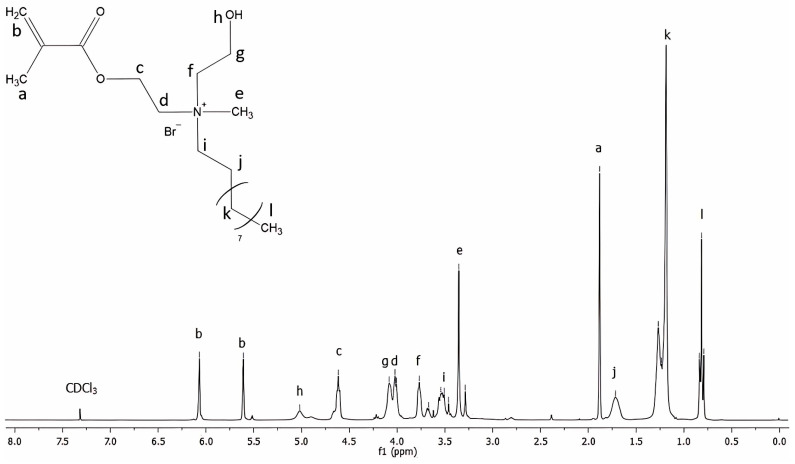
The ^1^H NMR spectrum of the QAHAMA-C10 monomer.

Figure 1 shows the ^1^H NMR spectrum of the QAHAMA-C10 monomer. Table 1 lists the proton signals presented in the spectrum with their assignment to chemical groups.

**Figure 2:**
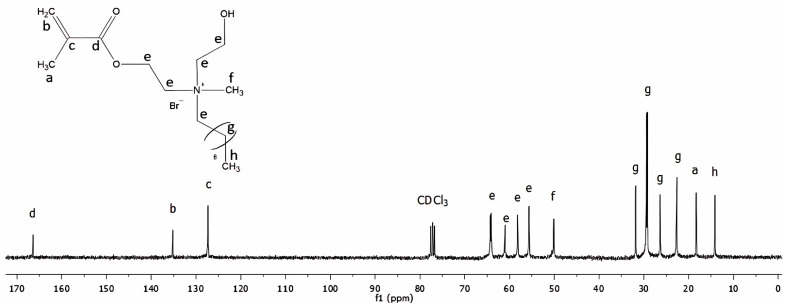
The ^13^C NMR spectrum of the QAHAMA-C10 monomer.

Figure 2 shows the ^13^C NMR spectrum of the QAHAMA-C10 monomer. In Table 2, the carbon atom signals presented in the spectrum, with their assignment to chemical groups, are listed.

### 2.2. Physico-Mechanical Properties

#### 2.2.1. Glass Transition Temperature and Thermal Decomposition

**Figure 3:**
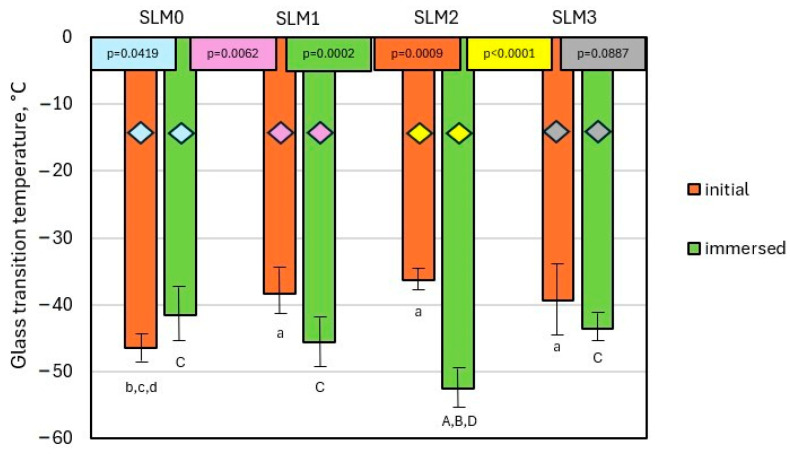
Mean glass transition temperature values with standard deviations for the SLMs; lowercase letters above the bars (a–d) and uppercase letters above the bars (A–D) indicate statistically different values with post hoc tests results (α = 0.05) and refer to subsequent bars; the values in the colored boxes correspond to the colors of the columns (ANOVA test) or correspond to the colored rhombuses on the columns (Student *t*-test).

Mean *Tg* values are shown in Figure 3. The mean *Tg* of the experimental SLMs before immersion in water, determined in the second heating run, were −38.33, −36.27, and −39.38 °C for SLM1, SLM2, and SLM3, respectively. All SLMs had a *Tg* higher than SLM0 (*Tg* = −46.43 °C). The *Tg*s of the SLMs determined after 28 days of their immersion in water were −45.73, −52.61, and −43.74 °C for SLM1, SLM2, and SLM3, respectively. SLM1 and SLM2 had *Tg*s lower than SLM0 (*Tg* = −41.63 °C), whereas SLM3 had a *Tg* similar to SLM0. For the SLMs before and after water immersion, there was a statistically significant difference (*p* = 0.0009 and *p* = 0.0002, respectively) between the groups. When comparing the same pairs of SLMs in the context of non-immersion and immersion in water, a statistically significant difference was found for SLM2 and SLM1 (*p* < 0.0001 and *p* = 0.0062, respectively). In contrast, for SLM0 and SLM3, no statistically significant differences were found (*p* = 0.0419 and *p* = 0.0887, respectively). At temperatures above 200 °C, several endothermic peaks were observed on the DSC thermograms. The first peak appeared in the thermogram of SLM0, with a minimum of 243 °C. The subsequent peaks, which occurred at higher temperatures, had minima ranging from 268 °C to 292 °C. Representative DSC thermograms for non-immersed and immersed-in-water SLMs are presented in Figure S2 in the Supplementary Materials.

#### 2.2.2. Shore a Hardness

**Figure 4:**
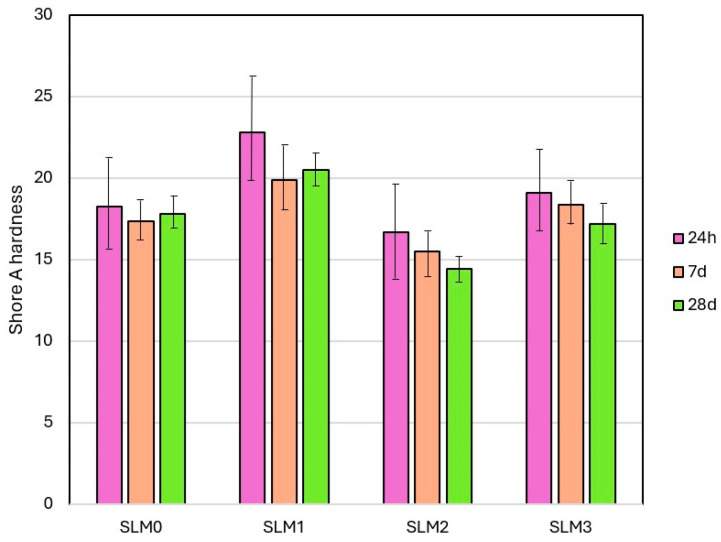
Mean hardness values with standard deviations for SLMs.

Mean *SHA* values are shown in Figure 4. Statistically significant differences in the hardness of different materials were registered (Table 3). The mean *SHA* values after 24 h of storage in distilled water ranged from 14.9 °Sh A(SLM2) to 22.8 °Sh A(SLM1), and the lowest value was registered for SLM2. After 7 days, the mean values were for SLM2 to SLM1 from 13.4 °Sh to 19.2 °Sh, respectively. After 28 days, the values were from 12.8 °Sh A (SLM2) to 20.5 °Sh A (SLM1), respectively. Significant changes in hardness (reduction) after storing were registered only for SLM1; for the other materials, the changes were not significant.

#### 2.2.3. Tensile Bond Strength of SLMs with Denture Base Material

**Figure 5:**
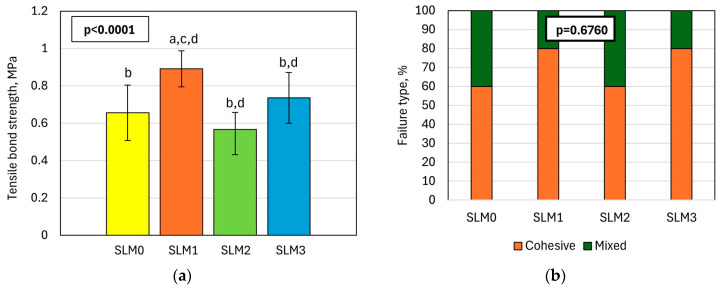
Results of the bond strength test with the denture base material test: (**a**) mean and standard deviations of bond strength; (**b**) on the type of fracture after bond strength tests, α = 0.05; lowercase letters above the bars (a–d) indicate statistically different values for (α = 0.05) and refer to subsequent bars.

The mean values of the *TBS* are shown in Figure 5a. The differences were statistically significant. The highest *TBS* was registered for SLM1 (0.89 MPa), and this value differed statistically from the results obtained for the other SLMs. Distributions by fracture type after bond strength tests are shown in Figure 5b. The dominant type was cohesive, but mixed have been registered. The material type has no significant influence on the distribution of fracture types (*p* = 0.6760).

#### 2.2.4. Tensile Strength of SLMs

**Figure 6:**
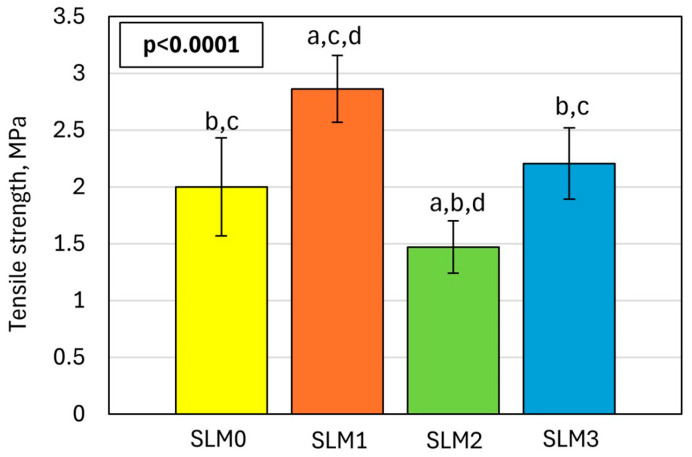
Mean tensile strength values with standard deviations for the SLMs; lowercase letters above the bars (a–d) indicate statistically different values (α = 0.05) and refer to subsequent bars.

The introduction of QAHAMA-C10 resulted in significant mean *TS* value changes (Figure 6); the highest values were registered for SLM1 (2.9 MPa), and the lowest forSLM2 (1.5 MPa).

#### 2.2.5. Water Sorption and Solubility

**Figure 7:**
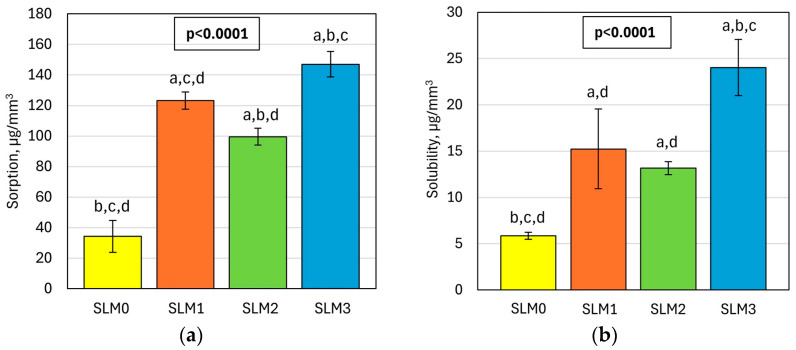
Mean sorption (**a**) and solubility (**b**) with standard deviations for tested SLMs; lowercase letters above the bars (a–d) indicate statistically different values (α = 0.05) and refer to subsequent bars; a is identical to the first bar, b is identical to the second bar, etc.

The mean values of *WS* and *WSL* are presented in Figure 7. The introduction of experimental monomer has a statistically significant influence on the *WS* and *WSL* values (*p* < 0.0001) of SLMs. Both properties’ mean values for the experimental materials were higher than for the control (34.4 µg/mm^3^ and 5.8 µg/mm^3^, respectively). The *WS* values were from 99.6 µg/mm^3^ to 147.1 µg/mm^3^ for SLM2 and SLM3, respectively, while the *WSL* ranged from 13.2 µg/mm^3^ to 24.0 µg/mm^3^ for SLM2 and SLM3, respectively.

### 2.3. Antifungal Properties

#### 2.3.1. Adherence of *Candida albicans* Cells

**Figure 8:**
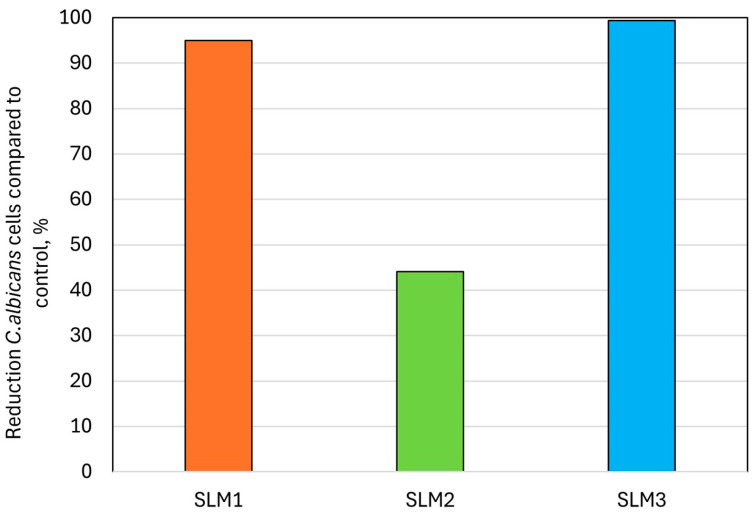
Reduction in *Candida albicans* cells compared to SLM0 (control).

For SLM1 and SLM3, the number of viable *C. albicans* cells adhered to the surface (Table 4) decreased significantly after the introduction of QAHAMA-C10, and the percentage reduction in comparison to SLM0 was 95% and 99% for SLM1 and SLM3, respectively (Figure 8).

#### 2.3.2. Cytotoxicity Test

**Figure 9:**
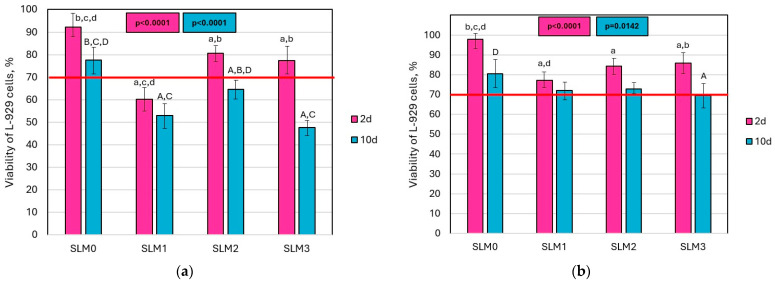
The viability of L-929 cells after 24 h of incubation with 2-day and 10-day undiluted (**a**) and two-times diluted (**b**) extracts. lowercase letters above the bars (a–d) and uppercase letters above the bars (A–D) indicate statistically different values (α = 0.05) and refer to subsequent bars; a is identical to the first bar, b is identical to the second bar, etc.

The mean values of L-929 cell viability for the extracts are shown in Figure 9. Significant differences (*p* < 0.0001) in the mean viability of L-929 cells were observed for the 2-day undiluted extracts. All extracts derived from the experimental materials resulted in decreased cell viability compared to the reference extract; however, only the SLM1 extract had average values below 70%. For the 10-day extracts, cell viability was significantly lower for all experimental materials compared to the control material, with all values falling below the 70% threshold. In contrast, the average cell viability for SLM0 was 77%. For the 2-day and 10-day two-times diluted extracts, significant differences were also noted (*p* < 0.0001 and *p* = 0.0142, respectively). The viability values remained above 70%, except for SLM3, which had a viability of 69%.

## 3. Discussion

Extensive research on SLMs has demonstrated that the incorporation of antifungal agents can produce effective antifungal activity against pathogenic yeast, such as *C. albicans*. However, it is essential to note that these studies demonstrated that most of the modified SLMs initially exhibited antifungal properties, which decreased with time. This is mainly due to the leaching of antifungal agents into the surrounding saliva, which decreases their concentration and effectiveness over time. It highlighted the demand for innovative research that would focus on the development of antifungal SLMs with improved stability and sustained antifungal activity. For this purpose, we used an antifungal agent in the form of a monomer containing quaternary ammonium groups that were capable of copolymerizing with methyl methacrylate. We expected that the covalent anchoring of QAGs in the SLM matrix would ensure the stability of their properties over a long time.

First, we determined the glass transition temperature of the soft lining materials, since this is a crucial property for their effective use. During the glass transition, the mechanical properties of the polymer change dramatically. Below the *Tg*, the material has a high hardness and elasticity modulus, while above the *Tg*, it becomes soft and highly elastic. Since SLMs act as a soft cushion that separates the hard denture plate from the tissues of the oral cavity, these materials must exhibit typical elastomeric properties. This includes a shock-absorbing effect, significant reversible deformation without fracturing, and a low hardness and modulus [43,44]. We conducted two types of DSC experiments: the first before water immersion, and the second after immersion in water for 28 days. For the first type of measurement, the *Tg* was determined from the second heating cycle, after initial heating up to 100 °C, which provided the inherent *Tg* of the material.

The *Tg* of the experimental SLMs before water storage ranged from −46.43 °C to −36.27 °C, comparable to the *Tg* obtained by Kitagawa et al. for acrylic SLMs [43]. An analysis of the *Tg* values showed that they increased according to the following order of tested materials: SLM3, SLM1, SLM2. A key observation was that an increase in the *Tg* values coincided with an increase in the plasticizer content. This finding suggests that the plasticizer plays a crucial role in influencing the *Tg* of the materials being studied. The storage of the SLMs in water resulted in a decrease in the *Tg* values, which ranged from −43.74 to −52.61 °C. These phenomena can be attributed to the plasticization of the methacrylate polymer by water molecules. It coincided with the findings of Lacoste-Ferré et al., who demonstrated that the water absorption of selected acrylic SLMs had a plasticization effect, which was manifested by a decrease in their modulus. They also showed that hydrated oral mucosa had a lower rigidity than dried mucosa [45]. Interestingly, the *Tg* of SLM0 increased after immersion in water. Such a difference in the behavior of the *Tg* between the experimental samples and the reference sample can be explained by the fact that the former contained QAHAMA-C10. Its quaternary ammonium groups are hydrophilic and therefore can promote the water absorption of a polymer. Finally, the highest *Tg* was observed for SLM3. It can be attributed to the highest concentration of EGDMA crosslinker, the content of which was almost twice as high in the case of SLM3 (3.1 wt.%) in comparison to SLM1 (1.78 wt.%) and SLM2 (1.82 wt.%). It suggests that the higher crosslink density in SLM3 had a greater impact on the *Tg* of water-immersed samples than the higher concentration of hydrophilic quaternary ammonium groups derived from QAHAMA-C10.

The DSC analysis revealed that the thermal decomposition of SLMs occurred at temperatures above 200 °C, primarily involving poly(methyl methacrylate) (PMMA) and DIOP. This decomposition was evidenced by the presence of endothermic peaks in the thermograms within that temperature range. In the thermograms of the experimental SLMs, one prominent peak was observed, with a minimum temperature ranging from 287.13 to 297.31 °C for dry samples and from 267.92 to 292.05 °C for water-immersed samples. Notably, the sample SLM0, which had the highest content of plasticizer, exhibited a peak at lower temperatures (240.20 °C for the dry sample and 242.92 °C for the water-immersed sample). These findings are consistent with the existing literature. According to Hu et al., PMMA begins to degrade at 220 °C, with significant mass loss, occurring between 220 °C and 270 °C, while complete degradation is achieved by 305 °C [46]. In turn, Fischer demonstrated that n-dioctyl phthalate, having a similar structure to the DIOP plasticizer that was used in our study, decomposes at 266 °C [47].

The hardness and its stability determine the proper functioning of the temporary SLMs. Their values depend on the content and type of plasticizer and solvent (the higher the content, the lower the initial value) and the chemical composition of the polymerizable ingredients of the materials. Ethanol is frequently incorporated into a wide range of SLMs at concentrations between 6% and 40 vol.%. It serves as a plasticizer and a solvent, enhancing the overall effectiveness of the SLM formulation. In our proposed SLM system, we assume that it will play the same role as above, but it also supports compatibility between QAHAMA-C10 and EMA. One of the key disadvantages of acrylate-based SLMs in contact with water in the environment of the oral cavity or during laboratory experiments is the leaching out the plasticizer, resulting in hardening [48]. During the current experiment for the control material, as well as for SLM2 and SLM3, the hardness values were stable over time. Additionally, these two materials also did not differ significantly in hardness from SLM0. Taking into account the fact that the percentage of plasticizer in SLM0 was almost 15% higher than in SLM3, it can be concluded that the high content of the large molecules of the developed QAHAMA-C10 monomer promoted hardness reduction, which is beneficial. At the same time, increased hardness values were observed for the SLM1 material compared to SLM0, which could be caused by the lack of EMA, the reduced plasticizer, and the limited percentage of QAHAMA-C10 in the liquid component. The slightly lower hardness values obtained for SLM2 could in turn be due to a higher plasticizer content compared to SLM1, with almost the same QAHAMA-C10 concentration. Storage in water was a reason for hardness only for SLM1; however, stabilization was reached after seven days. Some reductions in hardness were also observed for the remaining two experimental materials, although the differences were not statistically significant. This phenomenon can be related to the significantly higher water absorption of these materials compared to SLM0, because water molecules can penetrate the polymer chains and weaken their interactions, which is commonly indicated as a cause of the softening of acrylates [49,50]. So far, the influence of antimicrobial additives on changes in hardness has been noted. The results were varied; for example, some antibiotics, such as miconazole, chlorhexidine, and nystatin, can increase hardness, but others, such as ketoconazole or itraconazole, can decrease it [28,51,52,53,54]. However, previous results cannot be directly linked to the findings of the current investigations, because the antimicrobial monomers introduced into the polymer network of the acrylate soft dental soft linings were not tested before.

The bond strength of SLMs with denture base acrylates is one of the key properties that determine the functioning of the soft lining [55]. It is influenced by the chemical composition of the bonded materials, as well as the appropriate preparation of the hard acrylic surface [55]. The tensile bond strength test is the only standardized test, and although, considering the biomechanics of the prosthesis, it seems less justified compared to peal or shear tests, it is widely accepted due to the high repeatability of its results and its independence from the detailed conditions of the experiment [11]. It is assumed that the acceptable *TBS* values for materials with a hardness below 25 °Sh A, allowing long-term functionality, should be above 0.5 MPa [56]; however, some researchers suggest a value of 0.44 MPa as clinically satisfactory [57]; so, all the investigated materials were acceptable, similar to commercially used ones [58,59]. The registered cohesive percentage of mixed types of fractures were also recommended because they indicate a good quality of connection that allows for the proper use of strength [14]. *TS* values ranged from 1.5 to 2.8 MPa, which is typical for similar materials and shows the standard relation of *TS* to *TSB* [14]. The registered results should be considered beneficial, because after the introduction of antimicrobial agents such as nystatin, miconazole, ketoconazole, chlorhexidine, and itraconazole, the TS did not exceed 0.2 MPa [60]; when Kim et al. [61] modified acrylic SLM with nystatin and alginate microparticles, they did not note the effect of the modification.

The introduction of QAHAMA-C10 to the SLMs increased the *WS* and *WSL*. In contact with water or saliva, physically impermanently bound plasticizer and solvent molecules are leached out of the acrylic SLM system and replaced by an aqueous medium [62]. Although the ISO 10139-1:2016 standard [63] dedicated to T-SLMs does not require the determination of *WS* and *WSL*, these important properties were investigated due to their potential influence on mechanical properties and the amount of chemical ingredients that can enter the body with saliva. The disadvantage of the *WSL* and *WLS* test is the impossibility of clearly identifying the cause or chemical compound responsible for the changes that occurred. Despite these doubts, these tests prove to be useful in indicating the general nature of the changes that take place, and taking into account the precisely known chemical composition of the liquid components of the SLM used in the experimental materials, it is possible to indicate the possible cause of the registered changes.

It is most often emphasized that the loss of plasticizers is responsible for the increase in WS and WSL [64]. However, for the currently tested materials, this reason seems unlikely, because SLM0, with the highest DIOP content, showed the lowest values of the measured properties, while SLM3, with the lowest DIOP concentration, showed the highest values. This assumption is supported by the fact that for most materials the hardness values were stable over time, and the material with added QAHAMA-C10 decreased, while the loss of plasticizer should cause hardening. This indicates other causes of the registered changes. The QAHAMA-C10 monomer is a viscous resin that dissolves in EtOH, which justifies the composition of the addition of both ingredients to the LCs to improve the functional characteristics of the entire system. EtOH is commonly used in many commercial SLMs in amounts ranging from 6 to 40% by volume [65]. Due to the solubility of EtOH in water, there is a risk that unpolymerized QAHAMA-C10 dissolved in EtOH leaches out of the system relatively quickly and enters the water. However, the EtOH concentration can influence the amount and rate of release of other ingredients [65], so it is possible that its concentration can be operated to modify the QAHAMA-C10 release rate, if only to a limited extent. In our experiment, the influence of the EtOH concentration on the analyzed properties was visible (e.g., SLM1 vs. SLM2), but it was certainly not the main reason, considering the much higher WS and WSL values for SLM1 than for SLM0, despite the similar concentration of EtOH. The increase in the values of these properties with the increase in the concentration of QAHAMA-C10 was significant, indicating that its release into the environment was the cause of the observed changes. However, high values of WS and WSL are typical for temporary acrylic SLMs [48,62] and do not disqualify the material, if they do not harm its other properties, in particular the increase in the hardness after storing in water. For the investigated materials, such negative changes in hardness were not registered. It should be emphasized that in other works, an increase in the WS and WSL of acrylates was also observed as a result of the introduction of different antimicrobial additives [49,66].

Two modified materials show a significant reduction in the median adherence to *C. albicans,* which exceeded 95%. Tests were carried out on samples previously conditioned in water for one month to check their antimicrobial effectiveness after the allowed period of use. We deliberately refrained from testing the samples immediately after they were taken, because during this time acrylic SLMs may release their components into the environment, such as peroxides, stabilizers, or EtOH, which have antimicrobial properties [67,68]. The introduction of antimicrobial monomethacrylate monomer from QAG into the SLM system is an innovative idea not yet reported in the literature. The antifungal effect of similar monomers has been demonstrated in in vitro studies [41]. QAHAMA-C10 monomer was obtained by the Menschutkin reaction, which consists of the transformation of the tertiary amine of a monomethacrylate into a quaternary ammonium salt using an alkyl halide, in this case 1-bromodecane. This reaction allows for the controlled selection of the desired N-alkyl chain length. The structural elements of the QAHAMA-C10 monomer, such as the quaternary nitrogen atom, ten-carbon N-alkyl chain, and bromide cross-ion, influence its antimicrobial properties [69]. Selection of the appropriate N-alkyl chain length is very crucial to achieving the most effective antimicrobial activity [70,71]. Numerous studies indicate that with the increase in -CH_2_- groups in the N-alkyl chain, a maximum biocidal activity is reached at a certain point, which then decreases or disappears [72,73,74,75]. Gram-negative bacterial cells are surrounded by an additional cell membrane, which constitutes a protective barrier for them, making it more difficult for QAG monomers to act in the biocidal process compared to their effect on Gram-positive bacterial cells [76]. The mechanism of antimicrobial action of QAG compounds is based on electrostatic interactions between the positively charged nitrogen atom of the QAG structure and the negatively charged cell membrane of the microorganism. As a result of this interaction, QAG compounds can adsorb on the surface of the microorganism’s cell membrane, and the penetration of N-alkyl chains into the cell disrupts the integrity of the microorganism’s cell membrane and causes its rupture. The components that are key to its proper functioning are then released from its interior, along with calcium (Ca^2+^) and potassium (K^+^) ions, which leads to an increase in the osmotic pressure inside the cell and, consequently, to the shedding of its organelles and autolysis [77,78]. As mentioned above, the presence of EtOH was necessary to obtain complete homogenization for the sparingly soluble QAHAMA-C10 monomer. This may create some controversy as to whether the antifungal effect is not partly caused by the action of released EtOH. To verify this, an additional investigation was performed to confirm the SLMs’ lack of EtOH using ^1^H NMR. The spectra for all the SLMs (Supplementary Figure S1) confirm that no EtOH was detected in the water fraction. This clearly shows that the antifungal activities resulted from the introduction of QAHAMA-C10.

Despite the encouraging results for adherence, it should be remembered that we used a basic model test based on yeast-like fungi, so in the future, to confirm the effectiveness of the materials, it would be interesting to perform additional tests taking into account additional factors occurring in the oral cavity, such as the presence in mucin-containing artificial saliva and biofilm accumulation [79]. It should be borne in mind that the biofilm covering the material may limit to some extent its antimicrobial activity in the environment, although it is to be hoped that it will not become a reservoir of living microorganisms, as it is difficult to remove microorganisms adhered to the surface.

Cytotoxicity tests showed that the cell viability of L-929 mouse fibroblasts after using the 2-day undiluted extracts exceeded 70% for SLM0, SLM1, and SLM3, so that the materials do not show cytotoxicity in our experiment [80]. However, we decided to use a prolonged, two-day extraction time as the basic one in the study to increase the restrictiveness of the experiment, by taking into account the particularly high intensity of the release of components such as monomers and plasticizers from temporary SLMs during the first two days [81,82], and other facts, such that among denture wearers, 18.6% wear their dentures continuously, including during sleep, and 24.6% declare that they only sometimes remove them [83]. When the extraction time was extended to 10 days, all extracts of the experimental materials showed cytotoxicity. Cell viability was also lower for the experimental materials than for SLM0, which indicates that the introduction of monomer increases the cytotoxic potential, which may be important, especially when considering continuous use of the relined dentures. A few reports emphasize the importance of performing an in vivo biocompatibility test [53,84,85]. Hotta et al. evaluated the cytotoxicity of SLMs supplemented with nystatin, ketoconazole, and chlorhexidine diacetate in 60 male rats. They observed a negative effect of SLMs with the addition of ketoconazole on the thickness and surface of rat keratin [84]. Songsang et al. assessed the cell viability of human gingival fibroblasts in contact with three SLMs modified with nystatin and chlorhexidine gluconate. They did not report any cytotoxicity of the modified SLMs [53]. Zheng et al. demonstrated the cytotoxicity of nystatin against hamster cheek epithelial cells, but this was associated with the insolubility of nystatin in water [85]. Reductions in cell viability as a result of a prolonged extraction time were recorded for SLM1 and SLM2, which is consistent with other cytotoxicity studies of acrylic SLMs [86] and may be explained by an increase in the concentration of released components with a potential cytotoxic effect, such as a plasticizer [87], residual mono- and dimethacrylate monomers [88], and benzoyl peroxide (an initiator from the powder component of acrylic SLMs) [89].

## 4. Materials and Methods

### 4.1. Chemicals and Reagents

1-bromodecane, *N*-methyldiethanolamine (MDEA), methyl methacrylate (MMA), and ethyl methacrylate (EMA) were purchased from Acros Organics (Acros Organics, Geel, Belgium). Ethylene glycol dimethacrylate (EGDMA), diisooctyl phthalate (DIOP), N, N-dimethyl-p-toluidine (DMPT), and phenothiazine (PTZ) were purchased from Sigma-Aldrich (Sigma-Aldrich, St. Louis, MO, USA). Chloroform, potassium carbonate, and toluene were purchased from POCH S.A. (POCH S.A., Gliwice, Poland). Ethanol (ETOH) was purchased from Chempur (Chempur, Piekary Śląskie, Poland). Villacryl Soft powder was purchased from Zhermack (Zhermack, Badia Polesine, Italy). All reagents were used as received.

### 4.2. Monomer Synthesis and Characterization

**Figure 10:**
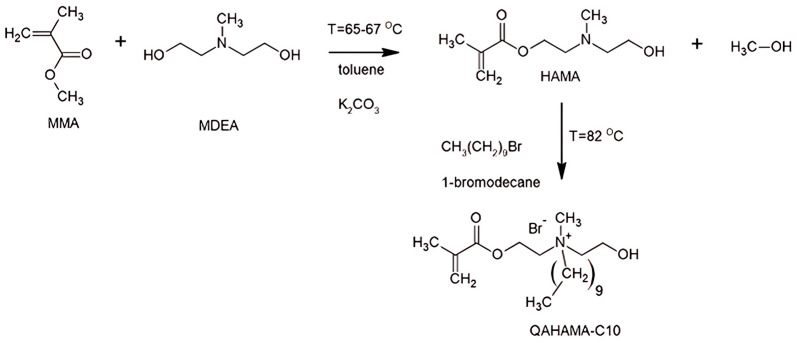
The synthesis route of the QAHAMA-C10 monomer.

QAHAMA-C10 was synthesized in a three-stage process (Figure 10) similar to that described by Chrószcz-Porębska et al. [40]. In the first step, MMA (1 mol, 100.12 g) was transesterificated with MDEA (0.67 mol, 79.85 g) in the presence of a reaction catalyst (K_2_CO_3_) (8 wt. %), a polymerization inhibitor (PTZ) (500 ppm), and toluene (407 mL). After the transesterification reaction, the reaction mixture was filtered from potassium carbonate and mixed with distilled water in a volume ratio of 2:1 on a magnetic stirrer. Then the mixture was separated in a separatory funnel by pouring the aqueous layers into a beaker. The process was repeated three times. The three aqueous layers collected in turn were mixed with chloroform in a volume ratio of 3:1, and this step was also repeated three times. The purified main product was vacuum distilled (2 mbar), taking a fraction at 120 °C. The obtained product, N, N-(2-hydroxyethyl)methylaminoethyl methacrylate (HAMA), had a yield of 19%. Next, the obtained pure HAMA (0.107 mol, 20.0 g) was then *N*-alkylated with a 1-bromodecane (0.107 mol, 23.67g) in the presence of PTZ (500 ppm) to avoid premature polymerization. The reaction was carried out with mechanical stirring at 82 °C for 5 days.

A Nuclear Magnetic Resonance Spectroscopy (NMR) 300 MHz spectrometer (UNITY/INOVA, Varian, Palo Alto, CA, USA) was used to collect 256 scans of ^1^H spectra and 40,000 scans of ^13^C NMR spectra of the QAHAMA-C10 achieved. CDCl_3_ was used as a solvent, and TMS was used as an internal standard.

### 4.3. Preparation of Experimental LCs

Three experimental liquid components were prepared by incorporating QAHAMA-C10, ETOH, EGDMA, and DIOP with the compositions and wt.% contents shown in Table 5 and were named LC1, LC2, and LC3, respectively. The control (LC0) was a liquid without QAHAMA-C10, but containing EMA instead. In formulating LC1, a constant molar ratio of QAHAMA-C10 to EGDMA of 7:3 was maintained. In LC2, the molar content of EtOH was reduced by half, and in LC3, the molar content of DIOP was reduced relative to the remaining LC components. The molar content was converted to the percentage of LCs.

### 4.4. Preparation of Soft Lining Materials and Their Polymerization

To each of the LCs was added 0.5 wt. % DMPT. The four SLMs were prepared by mixing LCs with the Villacryl Soft commercial powder component (PC) in a strict ratio of 2.2 g/1.6 mL (PC/LC) per 30 s. The pastes obtained were subjected to pressure polymerization at 65 °C for 30 min in a pressure polymerizer Palamat elite (Heraeus Kulzer, Hanau, Germany). Samples for tensile strength, microbiological, and immunological tests were cut from 2 mm thick polymerized plates polymerized in stainless steel molds. The remaining samples were made in stainless steel molds, ensuring the achievement of the intended shape and dimensions.

### 4.5. Physico-Mechanical Properties

#### 4.5.1. Glass Transition Temperature

Differential Scanning Calorimetry (DSC) measurements were performed with a heating rate of 10 K/min, in the air, using the DSC 3 (Mettler Toledo, Greifensee, Switzerland). SLM samples weighing approximately 2.5 mg were placed in aluminum crucibles. Measurements were carried out for the samples before water immersion (first run in the temperature range of −90 to 100 °C and second run in the temperature range of −90 to 300 °C), and after 28 days of immersion in water (experiments were performed in the temperature range of −90 to 300 °C). The glass transition temperature (*Tg*) was taken as the midpoint of the transition region. Its value was determined using Mettler Toledo STAR^e^ SW 16.30 (Mettler Toledo, Greifensee, Switzerland) software.

#### 4.5.2. Shore a Hardness

For Shore A hardness (*SHA*), the test methodology described in the ISO 10139-2:2016 [56] standard was used, with the use of additional detailed solutions resulting from the specificity of the material [14]. Five samples (40 mm in diameter, 6 mm in thickness) were polymerized for each material. Measurements were performed after 24 h, 7 days, and 28 days of conditioning in distilled water at 37 ± 1 °C. During the bath measurements, the temperature was kept constant by using stainless molds following the method previously described [14]. The hardness values were recorded at 5 points of every sample (indentation time was 5 s) with a digital durometer (Bareiss HPE II-A, Bariess, Oberdischingen, Germany). The mean value of the five measurements was the hardness of the sample.

#### 4.5.3. Tensile Bond Strength of SLMs with Acrylic

Tensile bond strength (*TBS*) to the PMMA denture base resin (DBR) was tested according to the ISO standard [56]. The surfaces of DBR plates (Vertex Castapres, Vertex-Dental B.V., Zeist, the Netherlands) measuring 15 mm × 15 mm and 4 ± 0.1 mm in thickness were wet-ground with P500-grit abrasive paper and conditioned in distilled water at 37 ± 1 °C for 28 days. Soft lining materials were placed on the stainless steel ring (a height of 3 mm and an internal diameter of 11 mm) placed between the DBR plates, polymerized, and conditioned in distilled water for 24 ± 1 h at 37 ± 1 °C; next, handles were mounted, and each sample was placed in the jaws of the universal testing machine (Zwick Z020, Zwick GmbH & Com, Ulm, Germany) and tensile tested until the break at a crosshead speed of 10 mm/min. The TBS (MPa) was calculated as a ratio of the maximal force (N) to the initial cross-sectional area (mm^2^).

Additionally, the type of fracture was classified as adhesive, cohesive, or mixed [14].

#### 4.5.4. Tensile Strength of SLMs with Acrylic

Ten samples of type 4 specified by the ISO 37 standard [90] were prepared for each material. The thickness and width were measured, and each sample was tensile tested (a crosshead speed of 10 mm/min) until the break using a universal testing machine; the ultimate *TS* (MPa) was the ratio of force at rupture (N) and the initial cross-sectional area (mm^2^).

#### 4.5.5. Sorption and Solubility

Sorption (*WS*) and solubility (*WSL*) were tested according to the ISO 10139-2:2016 standard [56]. Disc-shaped samples (50 mm in diameter, 0.5 mm in thickness) were dried to a constant mass (m_1_), and stored in distilled water for seven days at 37 ± 1 °C. The samples were removed from the water, surface dried, weighed (m_2_), and finally dried again to a constant mass (m_3_). The dryings were carried out at 37 ± 1 °C in a desiccator with freshly dried silica gel. All weightings were performed with an analytical balance (XP Balance, Mettler Toledo, Greifensee, Switzerland) of 0.0001 g accuracy. *WS* and *WSL* were calculated according to the following formulas:

(1) $$WS\;\left(\frac{µg}{mm^{3}}\right)=\frac{m_{2}-m_{3}}{V}$$

(2) $$SL\;\left(\frac{µg}{mm^{3}}\right)=\frac{m_{1}-m_{3}}{V}$$

where

*m*_1_—the initial mass of dried samples;
*m*_2_—the mass of swollen samples after a week of conditioning in water;
*m*_3_—the mass of dried samples after removal from water;
*V*—the initial volume of samples.

The results were averaged, and standard deviations (*SD*) were calculated for the samples.

### 4.6. Antifungal Properties

#### 4.6.1. Adherence of *Candida albicans* Cells

The adherence of *Candida albicans* cell test was performed with the previously described method [14]. The rectangular samples (10 × 10 × 2 mm) were conditioned in 300 ± 20 mL of distilled water for 30 days at 37 ± 1 °C, and the water was changed every 3 days. After that, dry and sterilized samples were placed for 18 h in 1 mL of *C. albicans* ATCC 10231 suspension ~1.5 × 10^5^ CFU/mL in tryptone water at 37 °C; next, they were vortexed in 1 mL of sterile water, and 100 µL of undiluted, obtained suspensions was seeded onto Sabouraud agar plates (bioMerieux) (Marcy l’Etoille, Lyon, France) and incubated at 37 °C for 24 h. The number of cells was determined by counting the colonies with a colony counter (ProtoCOL 3 PLUS, Synbiosis, Frederick, MD, USA). The percentage reduction of the mean values of *C. albicans* cells compared to the control group was calculated.

#### 4.6.2. Cytotoxicity—MTT Assay

The cell viability assay was performed based on the EN ISO 10993-5:2009 standard [80]. Polymerized samples that measured 10 × 10 × 2 mm were carefully rinsed (approximately one minute), dried, sterilized, and placed in 2 mL of culture medium used for the culture of fibroblasts of the L-929 line and incubated at 37 °C in an atmosphere of 5% CO_2_ for 2 or 10 days (2-day and 10-day extracts). The culture medium was incubated under the same conditions as a control. A suspension of cell culture of the L-929 line (NCTC clone 929) purchased from the American Type Culture Collection, catalog number CCL-1 (Manassas, VA, USA) was used, with a final density of 1 × 10^5^ cells/mL of medium. Finally, the viabilities of the L-929 cells contacted with extracts of the tested composites were evaluated. Mouse fibroblasts were incubated under in vitro culture conditions for 24 h with undiluted and two-times diluted extracts and their viability was assessed using the bromo-3-[4,5-dimethylthiazol-2-yl]-2,5-diphenyltetrazolium assay (MTT assay). DMSO was used to extract MTT formazan. The absorbance was determined at 550 nm using an Eon automatic plate reader (BioTek Instruments, Winooski, VT, USA). Cell viability (%) was calculated as a relation of the absorbance of the test sample to the absorbance of the control.

### 4.7. Statistical Analysis

Statistical analysis of the results was performed using PQStat version 1.6.6.204 (PQStat Software, Poznań, Poland). The residual distributions were tested with the Shapiro–Wilk test, and the equality of variances with the Levene test. The one-way with a possible F * correction (Brown–Forsythe) and Tukey’s HSD post hoc test or nonparametric Kruskal–Wallis test or Student *t*-test for pairs was used (α = 0.05). The impact materials used in the fracture observed in the bond strength test were tested with the exact Fisher–Freeman–Halton test for tables R × C (α = 0.05).

## 5. Conclusions

Satisfactory microbiological, immunological, and physico-mechanical properties were obtained for SLM3, for which almost total reductions of *C. albicans* were registered. Most of the SLMs investigated did not show cytotoxicity. Mechanical properties such as *SHA*, *TS,* and *TBS* were stable and satisfactory for this type of SLM. *WS* and *WSL* were at a high level; however, these properties do not constitute a qualification criterion from the point of view of clinical usefulness.

## Figures and Tables

**Table 1:** Signals characteristic of the QHAMA-C10 monomer in the ^1^H NMR spectrum.

Signal Symbol	Structure	Multiplicity	Number of Protons	Chemical Shift [ppm]
a	C**H_3_**-C=	s	3	1.92
b	=C**H_2_**	2m	2	5.65 and 6.10
c	-O-C**H_2_**-CH_2_-N^+^	m	2	4.65
d	-O-CH_2_-C**H_2_**-N^+^	m	2	4.06
e	C**H_3_**-N^+^	s	3	3.39
f	HO-C**H_2_**-CH_2_-N^+^	m	2	3.81
g	HO-CH_2_-C**H_2_**-N^+^	m	2	4.13
h	-O**H**	bs	1	5.08
i	N^+^-C**H_2_**-CH_2_-(CH_2_)_7_-CH_3_	m	2	3.59
j	N^+^-CH_2_- C**H_2_**-(CH_2_)_7_-CH_3_	m	2	1.75
k	N^+^-CH_2_- CH_2_-(C**H_2_**)_7_-CH_3_	m	14	1.22–1.30
l	N^+^-CH_2_- CH_2_-(CH_2_)_7_-C**H_3_**	t	3	0.87

**Table 2:** Signals characteristic of the QHAMA-C10 monomer in the ^13^C NMR spectrum.

**Signal Symbol**	**Structure**	**Chemical Shift [ppm]**
a	=C-**C**H_3_	19
b	>**C**=	136
c	**C**H_2_=	127
d	**C**=O	166
e	CH_3_-(CH_2_)_8_-**C**H_2_-N^+^	56–64
	-**C**H_2_-N^+^	
	-**C**H_2_-O-	
f	**C**H_3_-N^+^	51
g	CH_3_-(**C**H_2_)_8_-CH_2_-N^+^	23–32
h	**C**H3-(CH_2_)_8_-CH_2_-N^+^	14

**Table 3:** The results of one-way ANOVA and Tukey’s HSD post hoc tests for Shore A hardness *.

Material Type	Storage Time		
	24h (*p* < 0.0001)	7d (*p* = 0.002)	28d (*p* < 0.0001)
SLM0 (*p* = 0.324)	-; b,c	-;	-; b,c
SLM1 (*p* = 0.0071)	B,C; a,c,d	A;c	A; a,c,d
SLM2 (*p* = 0.0867)	-; b,d	-; b,d	-; a,b,d
SLM3 (*p* = 0.1432)	-; b,c	-;c	-; b,c

* Uppercase letters above for each row (A–C) and lowercase letters for each column (a–d) indicate statistically different values at the level of α = 0.05 and refer to subsequent columns or rows; e.g., a is identical to the first column, b is identical to the second column, etc.

**Table 4:** Antifungal efficacy for *Candida albicans* ATCC 10231 of the SLM and Kruskal–Wallis test results (α = 0.05) *.

Parameters of Descriptive Statistics	Vt, × 10 CFU/mL (*p* = 0.0007)			
	SLM0	SLM1	SLM2	SLM3
Med	752.5 ^b,d^	44.5 ^a,c^	438.5 ^b,d^	0 ^a,c^
Max	1060	54	636	16
Min	259	0	54	0
IQR	467.75	31.75	389.75	9

* Lowercase letters (a–d) indicate statistically different values (α = 0.0) and refer to subsequent medians in columns.

**Table 5:** Chemical compositions of LCs.

LC	EMA [wt.%]	QAHAMA-C10 [wt.%]	ETOH [wt.%]	EGDMA [wt.%]	DIOP [wt.%]
LC0 (control)	2.54	-	4.77	1.89	90.79
LC1	-	8.54	4.48	1.78	85.20
LC2	-	8.75	2.29	1.82	87.14
LC3	-	14.90	7.80	3.10	74.20

## Data Availability

The original contributions presented in this study are included in the article/Supplementary Material; further inquiries can be directed to the corresponding author/s.

## Supplementary Materials

The following supporting information can be downloaded at: https://www.mdpi.com/article/10.3390/molecules30040941/s1. Figure S1: The ^1^H NMR spectrum of the water fraction after storing SLMs in D_2_O: (a) SLM0; (b) SLM1; (c) SLM2; and (d) SLM3. Figure S2: Results of the DSC measurements: (a) thermograms of the second heating run of the samples before immersion in water; (b) thermograms of the second heating run of the samples after 28 days of immersion in water.
