# Supplementary material for: Effect of the Incorporation of an Innovative Monomer with a Quaternary Ammonium Group into a Temporary Soft Liner on Its Biological and Physicochemical Properties
Source: Molecules. 2025 Feb 18;30(4):941. doi: 10.3390/molecules30040941 (PMC11857937; doi:10.3390/molecules30040941)
Supplement: Supplementary file 1 [file molecules-30-00941-s001.zip › molecules-3410510-supplementary.pdf]

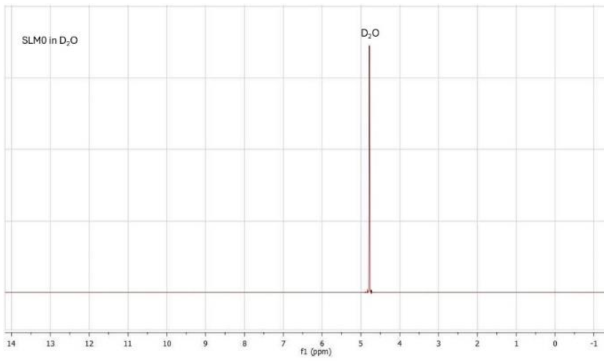

(**a**)

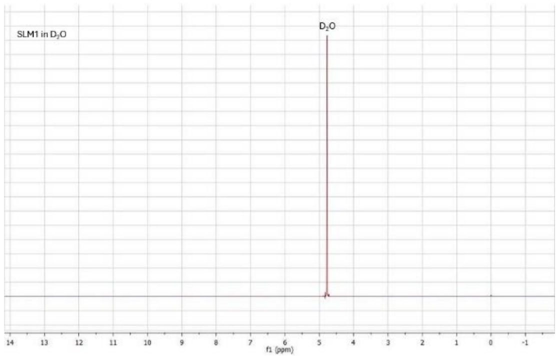

(**b**)

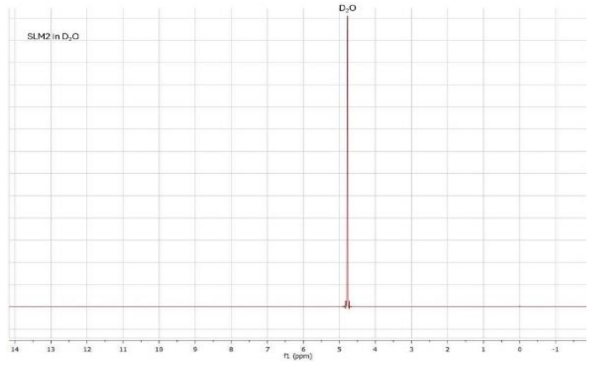

(**c**)

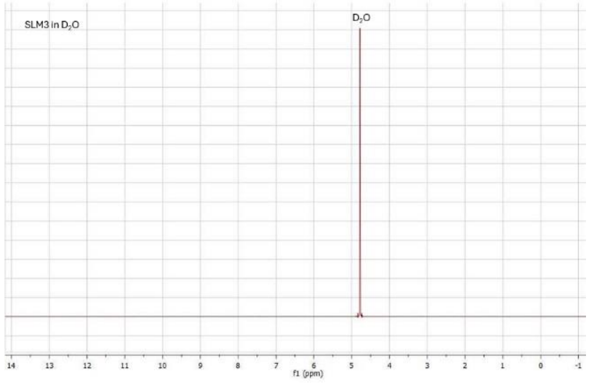

(**d**)

Figure S1. The ^{1}H NMR spectrum of the water fraction after storing SLMs in D_2O : (**a)** SLM0; (**b)** SLM1; (**c**) SLM2; and (**d**) SLM3.

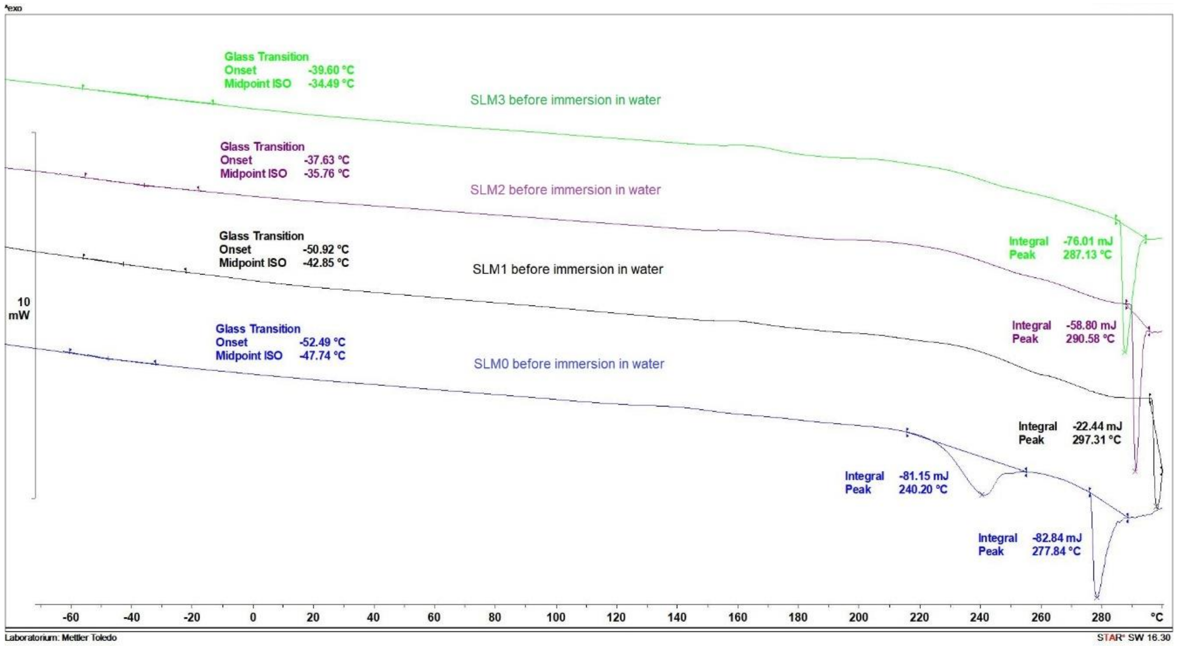

(**a**)

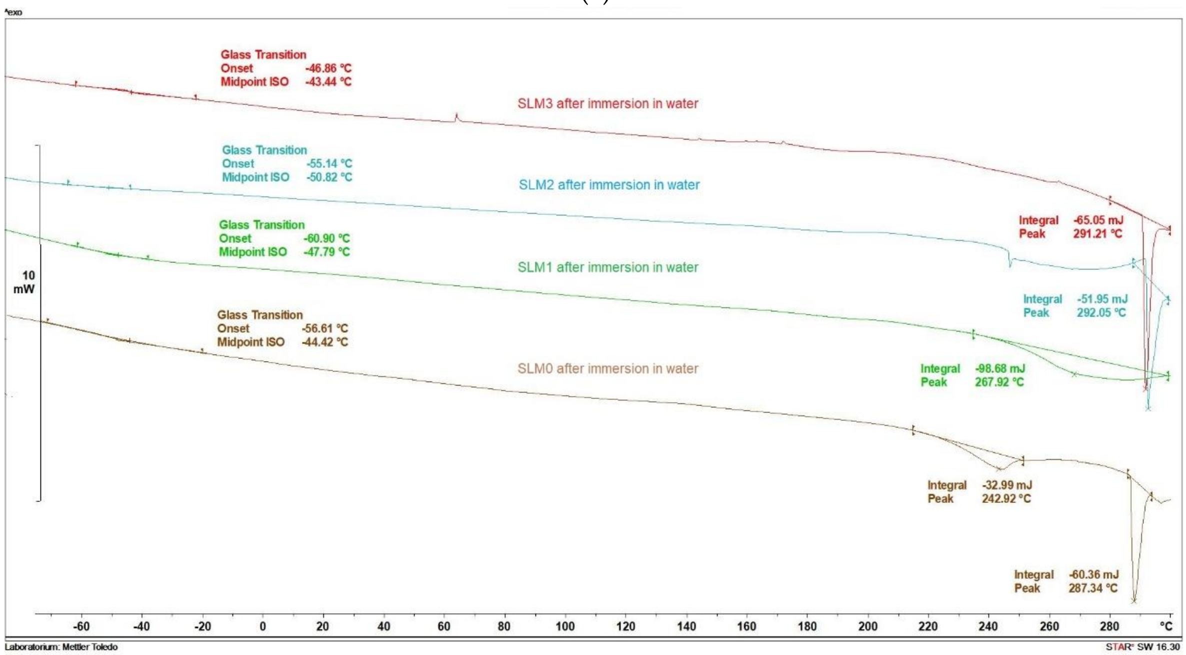

(b)

Figure S2. Results of the DSC measurements: (a) thermograms of the second heating run of the samples before immersion in water; (b) thermograms of the second heating run of the samples after 28 days of immersion in water.
